# Joint Early Stopping Criterions for Protograph LDPC Codes-Based JSCC System in Images Transmission

**DOI:** 10.3390/e23111392

**Published:** 2021-10-24

**Authors:** Zhiping Xu, Lin Wang, Shaohua Hong

**Affiliations:** 1 Department of Communication Engineering, Xiamen University, Xiamen 361005, China; xzpxmu@gmail.com (Z.X.); hongsh@xmu.edu.cn (S.H.); 2 National Institute for Data Science in Health and Medicine, Xiamen University, Xiamen 361005, China; 3 Shenzhen Research Institute of Xiamen University, Shenzhen 518057, China

**Keywords:** joint source-channel system, protograph LDPC, decoding, stopping criterion, cross entropy, image transmission

## Abstract

In this paper, a joint early stopping criterion based on cross entropy (CE), named joint CE criterion, is presented for double-protograph low-density parity-check (DP-LDPC) codes-based joint source-channel coding (JSCC) systems in images transmission to reduce the decoding complexity and decrease the decoding delay. The proposed early stopping criterion adopts the CE from the output likelihood ratios (LLRs) of the joint decoder. Moreover, a special phenomenon named asymmetry oscillation-like convergence (AOLC) in the changing process of CE is uncovered in the source decoder and channel decoder of this system meanwhile, and the proposed joint CE criterion can reduce the impact from the AOLC phenomenon. Comparing to the counterparts, the results show that the joint CE criterion can perform well in the decoding complexity and decoding latency in the low–moderate signal-to-noise ratio (SNR) region and achieve performance improvement in the high SNR region with appropriate parameters, which also demonstrates that this system with joint CE is a low-latency and low-power system.

## 1. Introduction

As the most challenging application scenario of the fifth-generation wireless communication, the ultra-reliable low-latency communications (URLLC) need to meet the latency requirement of 1 ms or less [1]. Though the methods of designing the short block length codes for URLLC [2] have been of concern, the architecture of the joint source and channel coding (JSCC) can also give another efficient way to satisfy the low-latency requirement. The JSCC system based on double protograph low-density parity-check (LDPC) codes [3,4,5,6,7,8,9,10,11,12,13,14] has been attracting increasing attention in recent years for its excellent properties, such as low power and low cost. In this system, protograph LDPC (P-LDPC) codes, which have the excellent properties of fast encoding speed and linear decoding implementation [15,16,17,18], are adopted to the source codes for compressing and channel codes for error correcting. In comparison to the JSCC system which is based on double LDPC codes [19], the double P-LDPC (DP-LDPC) JSCC system can obtain better performance both in waterfall region and lower error floor.

There have been many works focusing on the transmitting terminal of the DP-LDPC JSCC system, such as the coding design and its optimized methods. In the aspects of coding design, they are mainly from the perspectives of the source codes, channel codes, and connection edges, separately [3,4,5] or jointly [6,7,20]. By the use of the unequal power allocation method, the DP-LDPC JSCC system can obtain better performance [8]. An image preprocessing method is proposed for the DP-LDPC JSCC system to improve the transmission efficiency [9], and an adaptive image transmission method based on DP-LDPC system is proposed for the Internet of Things (IoT) scenarios [13]. The DP-LDPC JSCC system has also been applied to non-standard coding channels [10], including the fading channel with differential chaos shift keying modulation [11,12]. Though many works have done for the DP-LDPC JSCC system, the decoding-related works are less numerous, despite being very important for practical applications of this system’s implementation. In order to promote development of the DP-LDPC system to practical usage, a joint shuffled scheduling decoding algorithm is proposed for DP-LDPC JSCC system to reduce the decoding complexity and control the unequal convergence rated (UCR) phenomenon [14], it can achieve better performance and faster converged speed than joint belief-propagation (BP) algorithm.

The decoding complexity of LDPC codes can be approximately represented by $O\left(N*I\right)$ [21]. Here, *N* represents the number of the edges in LDPC codes, which is determined by coding design, and *I* represents the iteration number. To reduce the decoding complexity for the low-power applications, reducing the iteration number is a highly effective method, which is closely related to the decoding algorithms. The decoding algorithm mainly consists of the scheduling method and stopping criterion in the decoding process. Different from the purpose of scheduling methods to decrease the iteration numbers efficiently [14,21,22], early stopping criterions can avoid the unnecessary iterations in the low-moderate signal-to-noise ratio (SNR) region [23,24,25].

Stopping criterions determine whether the iterative process needs to continue. When the performance of the decoding process reaches a certain degree or the channel environment is bad, the further iteration process has little impact on the performance. Instead, unnecessary iterations will lead to higher decoding complexity and bigger delay, which are very harmful to the performance of DP-LDPC JSCC system. In order to tackle this problem, efficient early stopping criterions for DP-LDPC JSCC systems need to be discussed. There are different early stopping criterions in the tandem coding system that source coding and channel coding are separate. According to whether the a posteriori log-likelihood ratios (LLRs) of VNs are judged or not, they can be mainly classified into two kinds: soft decision-based (SDB) criterions [23,24,25] and hard decision-based (HDB) criterions [24,26,27]. So far, the existing stopping criterions are mostly designed for turbo codes and LDPC codes, which are used in separated source channel coding (SSCC) systems. Different from SSCC systems, these existing stopping criterions may need to be modified or redesigned while they are used in DP-LDPC JSCC systems.

In this paper, we design a joint CE stopping criterion for DP-LDPC JSCC system in image transmission, which considers the matching relationship between the source decoder and channel decoder. To be specific, we find the changing process of cross entropy (CE) in the JSCC system is different from that of the SSCC system. In summary, the main contributions of this paper are as follows.

(1) Early stopping criterion based on cross entropy is first introduced for the DP-LDPC JSCC system to avoid unnecessary iterations and the specific properties of the joint decoder are well considered. It can efficiently reduce the decoding complexity and lower decoding latency in low-moderate SNR region and achieve better performance in the high SNR region with appropriate parameters, compared to its counterparts.

(2) To overcome the impact from the oscillation-like phenomenon in the changing process of the CE in source decoder and channel decoder, which is unique in DP-LDPC JSCC system, unequal threshold factors are considered and the depth factor is adopted in the joint CE stopping criterion.

(3) It is found that the DP-LDPC JSCC system with joint CE stopping criterion can realize the trade-off between latency and performance, which is suitable for low-latency and low-power scenarios with appropriate parameters.

The reminder of this paper is organized as follows. Section 2 briefly describes the image transmission system model, the DP-LDPC system, and the process of the joint BP algorithm. The proposed early stopping criterions for DP-LDPC system are detailed in Section 3. The simulation results are presented and analyzed in Section 4. Finally, Section 5 concludes this paper.

## 2. System Model

### 2.1. Image Transmission System Model

**Figure 1:**
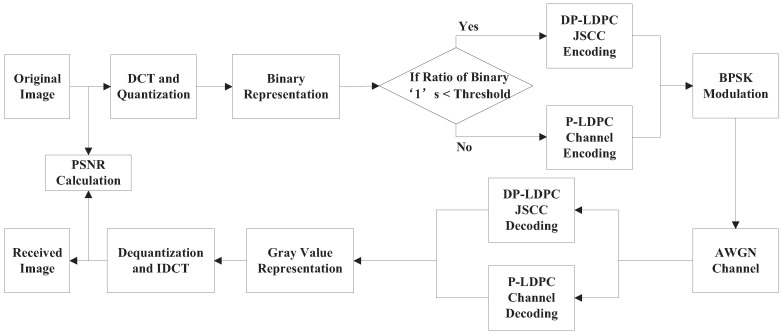
The image transmission system model.

Figure 1 shows the image transmission system model based on the DP-LDPC JSCC system. When the DP-LDPC JSCC system is applied to image transmission, the entropy of the original images is an important factor which impacts the performance. When the original images are represented by binary bit streams, the entropy of the bit steams is too large and cannot be used. Therefore, the discrete cosine transform (DCT) on the original images is performed and then the DCT coefficients are quantized. After these two operations, the original images which represented by binary bit streams are divided into equal length frames. As these frames have different statistical properties, a probability threshold is adopted to classify these frames. If the probability of binary ‘1’ of one frame is less than this threshold, this frame will be processed by the DP-LDPC JSCC system; otherwise, it will be processed by P-LDPC channel codes without source coding. After JSCC encoding or only channel encoding, the codeword is transmitted in additive white Gaussian noise (AWGN) channel by binary phase shift keying (BPSK) modulation. At the receiver, the received signals are decoded by the joint source channel decoder (JSCD) or only the channel decoder. The decoded binary bit streams are used to calculate the gray values of the received image, and the operation of inverse DCT (IDCT) is performed to recover the original image.

### 2.2. DP-LDPC JSCC System Model

**Figure 2:**
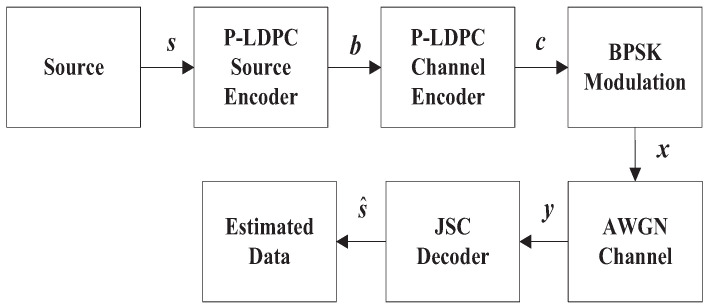
The DP-LDPC system model.

The model of the DP-LDPC JSCC system is shown in Figure 2. At the transmitter of DP-LDPC JSCC system, the sequence of information bits **s** is compressed by an unpunctured P-LDPC code at first, and then the sequence of compressed bits **b** is protected by another punctured P-LDPC code. Afterwards, the sequence of encoded bits **c** is transmitted in AWGN channel by BPSK modulation as the sequence of **x**. At the receiver, the JSCD is used to recover the sequence of the corrupted information bits **y** by joint BP decoding algorithm. The information bits **s** are generated by a binary independent and identically distributed (i.i.d.) Bernoulli source with entropy

(1) $$H\left(s\right)=-p\log_{2}\left(p\right)-\left(1-p\right)\log_{2}\left(1-p\right)$$

where $p=\mathrm{Pr}\left(s_{i}=1\right)$ and p≠0.5.

**Figure 3:**
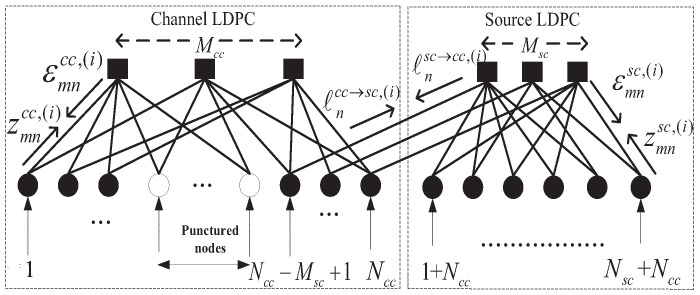
Joint decoder of the DP-LDPC system.

Figure 3 shows the joint decoder of the DP-LDPC JSCC system with a joint Tanner graph. The joint decoder works parallelly and exchanges the messages between these two decoders along the edges between variable nodes of channel decoder and the check nodes of source decoder with the joint BP algorithm. In this paper, the SNR means the ratio of the bit energy $E_{b}$ to the noise spectral density $N_{0}$, that is $E_{b}/N_{0}$.

### 2.3. Joint BP Algorithm

The joint-BP decoding algorithm is described shortly as follows. For the sake of simplicity, there are six types of LLRs are shown in Figure 3 and defined in [14]. $H_{cc}$ and $H_{sc}$ represent the parity check matrices of the channel protograph and source protograph, respectively. The maximum number of decoding iterations is set to $I_{\max}$. We assume an AWGN channel with zero mean and variance $\sigma^{2}$. The initialization process is the same as the process in [14]. After the initialization process, the main process of joint BP decoding algorithm is as follow:

***Step 1:*** LLRs updating.

***Channel decoder:***

For $1\leq n\leq N_{cc}$ and $1\leq m\leq M_{cc}$, calculate $\varepsilon_{mn}^{cc,(i)}$ and $z_{mn}^{cc,(i)}$.

For $N_{cc}-M_{sc}+1\leq n\leq N_{cc}$ and each m∈M(n), calculate $\ell_{n}^{cc\to sc,(i)}$.

***Source decoder:***

For $N_{cc}+1\leq n\leq N_{cc}+N_{sc}$ and $1\leq m\leq M_{sc}$, calculate $\varepsilon_{mn}^{sc,(i)}$ and $z_{mn}^{sc,(i)}$.

For $1+N_{cc}\leq n\leq N_{sc}+N_{cc}$ and each m∈M(n), calculate $\ell_{n}^{sc\to cc,(i)}$

***Step 2:*** Hard decision.

Calculate $z_{n}^{cc,(i)}$ and $z_{n}^{sc,(i)}$, and obtain the $\hat{c}$ and $\hat{s}$.

***Step 3:*** Stopping Criterion.

If $H_{sc}\hat{s}=\hat{c}$$H_{sc}\hat{0}=0$ and $H_{cc}\hat{c}=0$ are both satisfied, or $i=I_{\max}$, the iteration will be stopped and go to Step 4. If the stopping criterions are not met, set i=i+1 and go to Step 1.

***Step 4:*** Output $\hat{s}$ as the decoded source sequence.

## 3. The Proposed Stopping Criterions

Based on the works in [23,24,25], the *i*-th iteration is considered in the joint decoder. We first extend the HDA stopping criterion to the DP-LDPC JSCC system directly and then the proposed joint CE, which combines the properties of this system is detailed in the following.

### 3.1. Joint HDA Criterion

The HDA criterion can be applied to the DP-LDPC JSCC system directly:

(1) Compute the hard decision of the source decoder and channel decoder, $D_{sc}^{i}\left(j\right)$ and $D_{cc}^{i}\left(j\right)$, of the source decoder and the channel decoder LLRs for the j-th bit $z_{n}^{sc,(i)}$ and $z_{n}^{cc,(i)}$.

(2) If $D_{sc}^{i}\left(j\right)=D_{sc}^{i-1}\left(j\right)$ and $D_{cc}^{i}\left(j\right)=D_{cc}^{i-1}\left(j\right)$ are satisfied for all VNs in both decoders, or $i=I_{\max}$, the iteration will stop. Otherwise, continue with the iteration.

### 3.2. Joint Cross Entropy Criterion

The cross entropy is defined as the difference of the output LLRs between the current iteration and last iteration in the source decoder and channel decoder; the cross entropy of both decoders are represented by $CE_{sc}\left(i\right)$ and $CE_{cc}\left(i\right)$, respectively. The source decoder will stop the iteration when the value of its CE drops to a certain threshold, which means that performance will not improve with more iterations. The channel decoder is the same as the source decoder.

**Figure 4:**
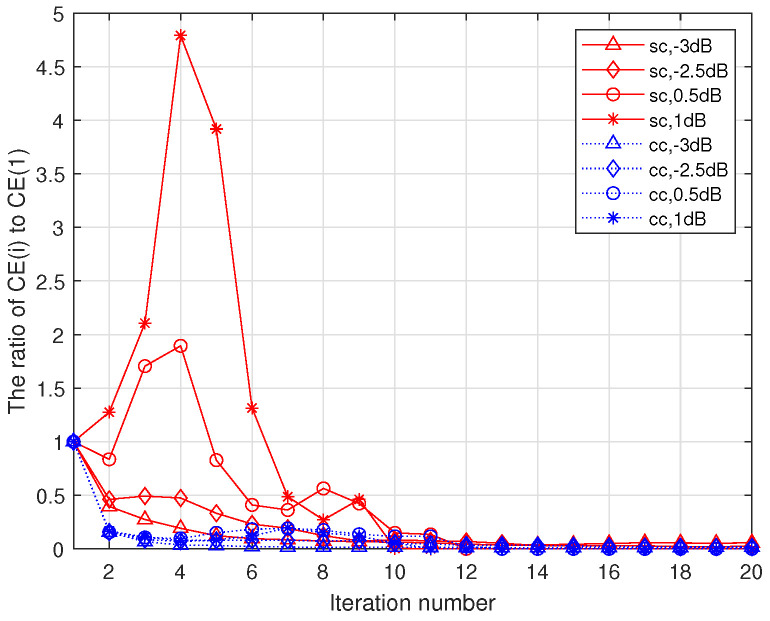
The ratio of cross entropy at the *i*-th iteration and cross entropy at the first iteration versus the decoding iteration numbers for the source decoder (solid curves) and channel decoder (dashed curves) at different $E_{b}/N_{0}$.

Figure 4 shows the changing process of the ratio of the cross entropy at the *i*-th iteration (denoted by CE(i)) to the cross entropy at 1-th iteration (denoted by CE(1)) in the different decoders. For the source decoder, the ratio can be calculated by $\frac{CE_{sc}\left(i\right)}{CE_{sc}\left(1\right)}$. For the channel decoder, the ratio can be calculated by $\frac{CE_{cc}\left(i\right)}{CE_{cc}\left(1\right)}$. Figure 4 shows the changing process of the ratio of CE and CE at the first iteration with the increasing of the iteration numbers for source decoder and channel decoder. We can see that the ratios stably decreases during the iteration process in the low-moderate SNR region for both decoders, but the ratio of source decoder will rise by a large margin first and decrease later in the high SNR region. This phenomenon also exists in channel decoder, but it is not obvious. The oscillation phenomenon in DP-LDPC JSCC system, which we call asymmetry oscillation-like convergence (AOLC), is unique in this system and different from SSCC system, whose ratio stably decreases in the whole SNR region. In order to solve the problem from AOLC, we also introduce the depth factor represented by D. This depth factor can reflect whether the decoding process is stable, the details of the joint CE algorithm are as follows:

(1) Set the threshold factor α and β for source decoder and channel decoder respectively and reset the count *C*.

(2) At the end of the *i*-th (i≥1) iteration, compute

(2) $$CE_{sc}\left(i\right)=\sum_{j=1}^{N_{sc}}\left[\frac{{\left|L_{sc}^{i}\left(j\right)-L_{sc}^{i-1}\left(j\right)\right|}^{2}}{\exp\left(\left|L_{sc}^{i-1}\left(j\right)\right|\right)}\right]$$

(3) $$CE_{cc}\left(i\right)=\sum_{j=1}^{N_{cc}}\left[\frac{{\left|L_{cc}^{i}\left(j\right)-L_{cc}^{i-1}\left(j\right)\right|}^{2}}{\exp\left(\left|L_{cc}^{i-1}\left(j\right)\right|\right)}\right]$$

(3) If $CE_{sc}\left(i\right)<$ $\alpha *CE_{sc}\left(1\right)$ and $\;CE_{cc}\left(i\right)<$ $\beta *CE_{cc}\left(1\right)$ are satisfied, C=C+1.

(4) If C=D or $i=I_{\max}$, the iteration will stop. Otherwise, the iteration will continue.

## 4. Simulation Results

In this section, the simulation results consist of three parts: performance analysis of the joint CE algorithm, performance between joint CE and other stopping criterions, and image transmission performance comparison. In all the simulations, the frame length is set as 3200 bits and the maximum iteration number is set as 50. The simulations are assumed under an AWGN channel and all encoded bits are modulated by BPSK. The rate-1/2 $Bs_{1}$ code is used as the source code and the rate-1/2 $Bc_{1}$ codes is used as the channel code [5], which means overall rate is 1. Their base matrices are given as follows.

(4) $$Bs_{1}=\left(\begin{array}{cccccccc} 3 & 3 & 1 & 0 & 4 & 1 & 0 & 0\\ 4 & 3 & 0 & 1 & 5 & 1 & 1 & 0\\ 4 & 2 & 0 & 2 & 5 & 0 & 1 & 1\\ 4 & 3 & 2 & 0 & 5 & 0 & 0 & 1 \end{array}\right),Bc_{1}=\left(\begin{array}{ccccc} 1 & 0 & 1 & 0 & 0\\ 0 & 1 & 2 & 1 & 1\\ 0 & 1 & 1 & 2 & 2 \end{array}\right)$$

The label Original represents the traditional method to stop the iteration in joint BP algorithm, that is, the parity-check constraints of the both decoders are satisfied or the iteration number reaches $I_{max}$. The label (α,β) in figures means the threshold factors in both decoders, for example, the label (0.1,0.0001) means the threshold factor for source decoder is 0.1 and the threshold factor for channel decoder is 0.0001.

### 4.1. Performance Analysis of Joint CE Algorithm

**Figure 5:**
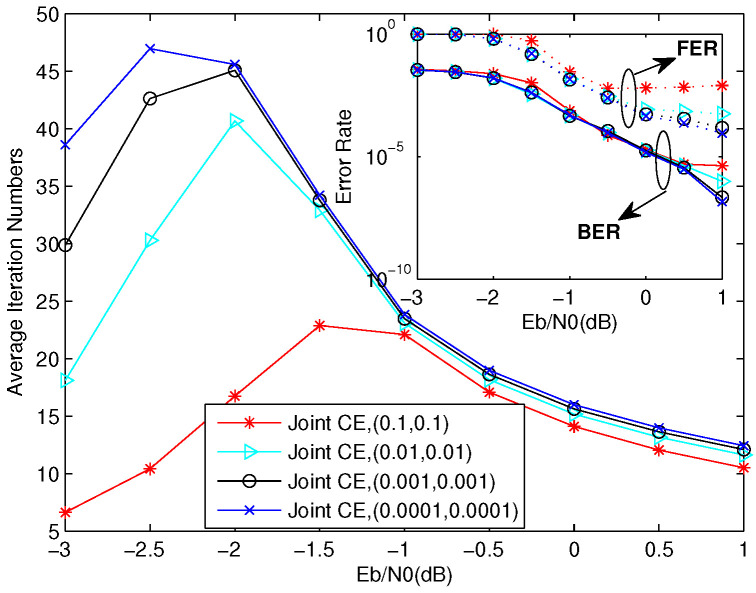
BER, FER, and convergence performance of joint CE in different threshold factors when α=β.

Figure 5 shows the bit error rate (BER), frame error rate (FER), and convergence performance of joint CE stopping criterions using different threshold factors and both decoders have the same threshold factor, which is α=β. With the threshold factor decreasing, we can see that the BER performance is nearly the same in the low–moderate SNR region, and the FER performance has the same situation as the BER performance, but FER gradually improves in the high SNR region. We can also find that the iteration number increases in the low–moderate SNR region with the threshold factor decreasing, and it nearly the same in the high SNR region.

**Figure 6:**
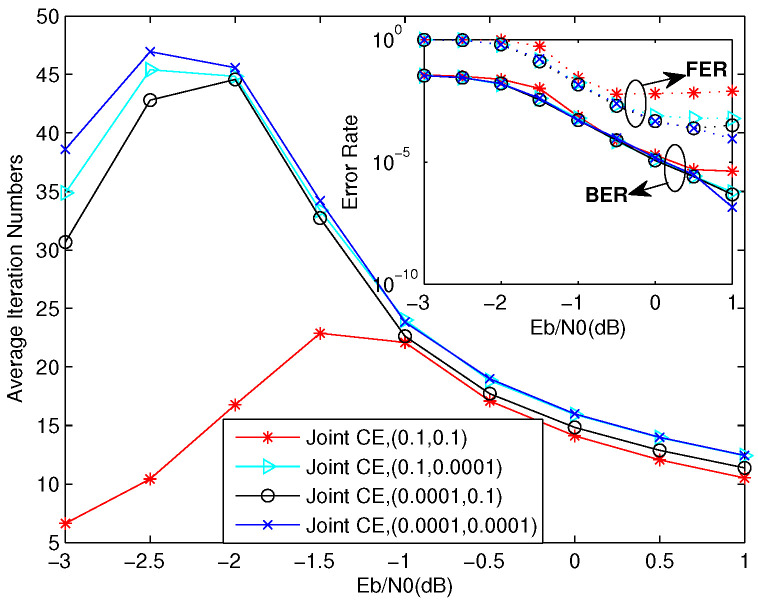
BER, FER, and convergence performance of joint CE in different threshold factors when one threshold factor is fixed and the other threshold factor is changed.

Figure 6 shows the BER, FER, and convergence performance of joint CE stopping criterions using different threshold factors and both decoders have the different threshold factor when one threshold factor is fixed and the other threshold factor is changed. While the threshold factor of source decoder α remains unchanged, decreasing the threshold factor of channel decoder β improves the BER and FER performance, and reduce the convergence speed. With the value of α decreasing, the effects of changing β decreases. While β remains unchanged, decreasing α can nearly bring the same effects.

**Figure 7:**
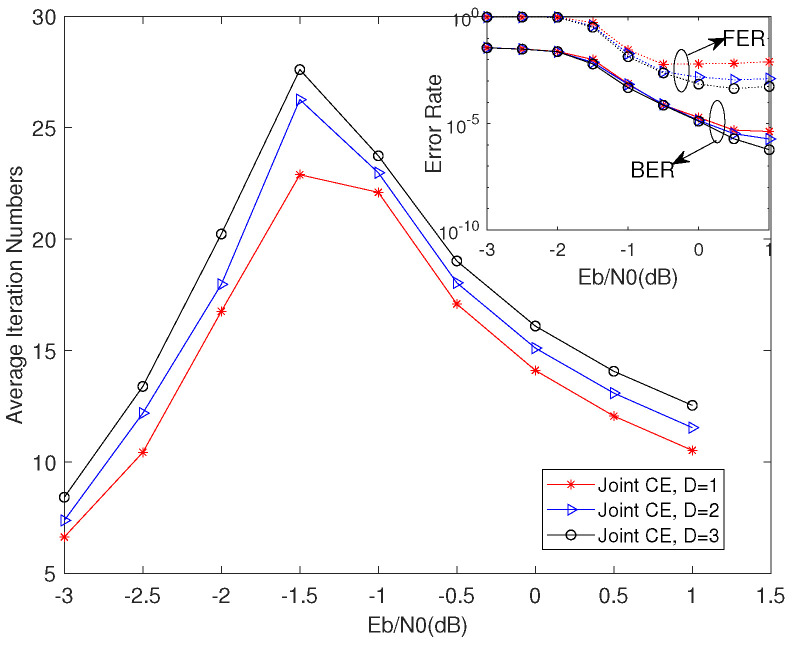
BER, FER, and convergence performance of joint CE in different depth factor at the same threshold factors when α=β.

From Figure 7, we can infer the effects of depth factor D on BER, FER, and convergence in the same threshold factors. With the value of the depth factor to further increase, it is quite clear that the BER performance improves slightly, and the improvements seen in the FER performance were greater. We can also see that the iteration number increases with the depth factor to further increase while joint CE stopping criterions can still keep the low iteration number in the low-moderate SNR region and bring the extra iterations in the high SNR region. Above all, we can summarize the properties of the joint CE stopping criterions as follows:

(1) The higher threshold factors, the faster convergence, worse FER performance, and little influence on BER performance with safe property in low–moderate SNR region.
(2) The depth factor can obviously improve the FER performance, improve BER performance slightly, and lower the converged speed when this factor increases.

### 4.2. Performance Comparison

**Figure 8:**
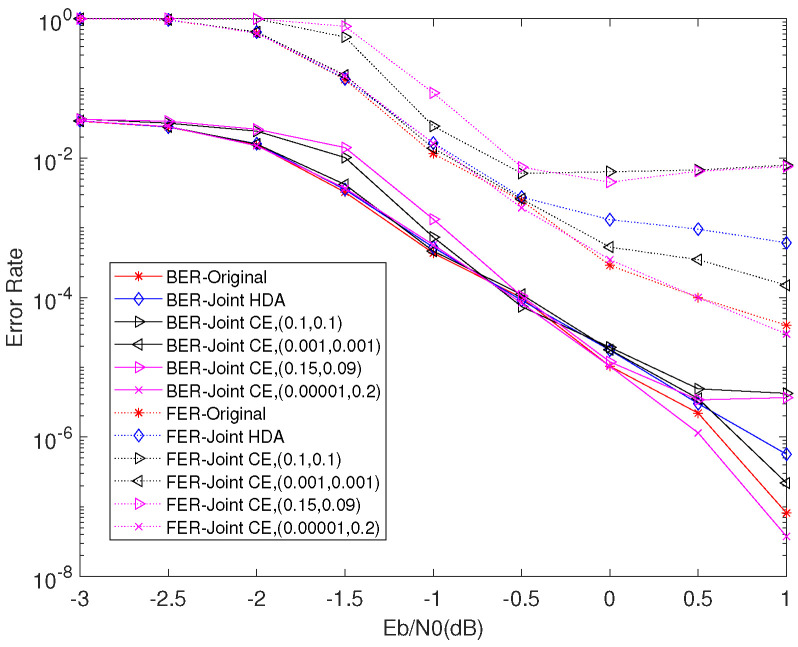
BER and FER performance of Original, joint HDA, and joint CE with different threshold factors.

Figure 8 shows the BER and FER performance of Original, joint HDA, and joint CE algorithm under p = 0.04. We can see that the BER performance of joint CE methods with appropriate parameters are nearly the same as the Original and joint HDA algorithm before the BER level at $1\times 10^{-4}$. At the FER = $2\times 10^{-3}$, the FER performance of the joint CE with α=0.001 and β=0.001 is nearly the same as the Original and outperforms HDA by 0.3 dB. Furthermore, the joint CE with α=0.00001 and β=0.2 can even obtain 0.1dB gain at BER = $1\times 10^{-6}$, comparing to the Original.

**Figure 9:**
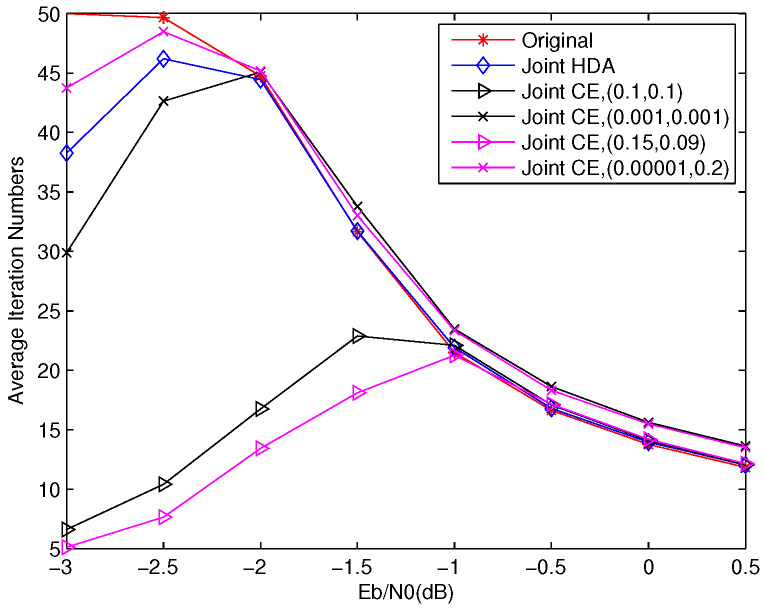
The comparison of convergence among Original, joint HDA, and joint CE with different threshold factors.

Figure 9 plots the average number of iterations (AINs) versus $E_{b}/N_{0}$ with *p* = 0.04. We can easily find that the proposed stopping criterions, including the joint CE and joint HDA algorithm, can efficiently reduce the AINs in low–moderate SNR region. For example, the AINs of joint CE, with α=0.001 and β=0.001, is 30% and 40% lower than Original and joint HDA at $E_{b}/N_{0}=-3\;dB$. Moreover, the joint CE with α=0.15 and β=0.09 can converge faster than the joint CE with α=0.1 and β=0.1. It is different from the SSCC system, whose converge speed is just decided by only one threshold factor and proportional to the value of threshold factor. This phenomenon further demonstrates that the joint CE with unequal thresholds can achieve better performance. We can also see that joint CE algorithm can obtain safe properties in BER and FER, and reduce the AINs in low–moderate SNR region. Through adopting appropriate parameters, it can also achieve the same AINs as the Original in the high SNR region.

From Figure 5, Figure 6 and Figure 7 and Figure 9, we can summarize the properties of the DP-LDPC system with the joint CE as follows:

(1) In the low–moderate SNR region, this system with joint CE can realize low latency with a little performance loss and the AINs are lower than 25 (half of the value of the maximum iteration), which can satisfy the requirement of the voice transmission at the BER level of $10^{-3}$ with low latency property.

(2) In the high SNR region (larger than 0 dB), this system with joint CE can realize lower latency with appropriate parameters to satisfy the requirement of the wireless communications at the BER level of $10^{-6}$. Note that the AINs of this system are less than 15 when the SNR is larger than 0 dB, while the SSCC system cannot work well when considering the same conditions.

(3) The DP-LDPC system with joint CE is a low-latency and low-power transmission system.

### 4.3. Images Transmission Comparisons

To verify the applicability and feasibility of the proposed joint early stopping in specific application, we consider the application of our proposed technique to image transmission that uses an equal error protection technique [9]. To evaluate the performance of the images transmission system, there are many quality assessment methods [9,28,29], and we adopt the peak signal-to-noise ratio (PSNR) to describe the quality of recovered images objectively [9]. The formula of PSNR computing is defined as

(5) $$PSNR=10\times \mathrm{lg}\frac{D^{2}\times M\times N}{\sum_{x=1}^{M}\sum_{y=1}^{N}{\left(I_{T}\left(x,y\right)-I_{R}\left(x,y\right)\right)}^{2}}$$

where the size of transmitted image is M×N, and $I_{T}\left(x,y\right)$ and $I_{R}\left(x,y\right)$ represent the original image and the recovered image, respectively. The value of *D* is 255, which is the peak value of signals for gray images.

Table 1 shows the AINs of different stopping criterions when PSNR values are evaluated at $E_{b}/N_{0}$ = − 2.5 dB. From this table, we can find that the AIN of the joint CE with α=0.15 and β=0.09 (denoted as “joint CE2”) is 38% and 36% lower than the Original and joint HDA algorithm with a little performance loss, and the joint CE with α=0.0001 and β=0.02 (denoted as “joint CE1”) can achieve the same AIN as the Original algorithm with nearly the same PSNR value.

**Figure 10:**
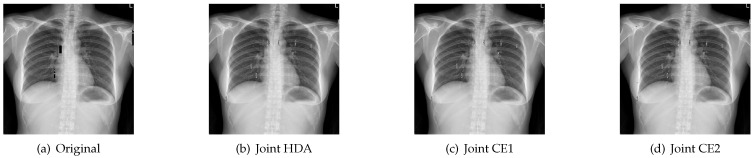
Reconstructed images with different stopping criterions for “X-ray” when $E_{b}/N_{0}$ = 1.5 dB.

**Figure 11:**
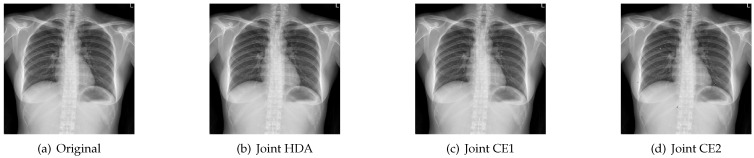
Reconstructed images with different stopping criterions for “X-ray” when $E_{b}/N_{0}$ = 2 dB.

Figure 10 and Figure 11 show the reconstructed images with different stopping criterions for “X-ray” when $E_{b}/N_{0}$ are 1.5 dB and 2 dB. It can be seen that the received images with the joint CE algorithm can be recovered with the same good quality as the original algorithm in the high SNR region. Table 2 shows the PSNR values of different stopping criterions at 1.5 dB and 2 dB. From this table, we can see that the joint CE1 can achieve the same PSNR value as the Original at $E_{b}/N_{0}$ = 2 dB, and has a lower PSNR value than the Original at $E_{b}/N_{0}$ = 1.5 dB.

## 5. Conclusions

In this paper, we proposed early stopping criterions based on LLRs values, including joint HDA and joint CE algorithms to reduce the unnecessary iteration numbers for the DP-LDPC JSCC system to decrease delay in low-moderate SNR region and achieve the performance improvement in the high region for the image transmission. By introducing the unequal thresholds factors and the depth factor, the joint CE can efficiently utilize the asymmetry oscillation-like convergence (AOLC) phenomenon, which does not appear in tandem coding system. Through these works, it is known that this system with joint CE can realize the trade-off between latency and performance, which can be applied to different application scenarios, and it is found that this system with the joint CE is a low-latency and low-power system. In the future, we will adopt the unequal error protection method to further improving better images transmission performances.

## Figures and Tables

**Table 1:** Comparison of AINs of different stopping criterions when PSNR values are evaluated at $E_{b}/N_{0}$ = −2.5 dB.

Algorithm	Original	Joint HDA	Joint CE1	Joint CE2
AIN	30.35	29.45	30.35	18.75
PSNR	10.9994	10.9927	10.9991	9.3823

**Table 2:** Comparison of PSNR values of different stopping criterions at different $E_{b}/N_{0}$.

$E_{b}/N_{0}$	Original	Joint HDA	Joint CE1	Joint CE2
1.5dB	30.0221	28.9290	28.9294	28.7658
2dB	38.0900	38.0834	38.0900	35.5038
